# Hierarchical Interleaved Bloom Filter: enabling ultrafast, approximate sequence queries

**DOI:** 10.1186/s13059-023-02971-4

**Published:** 2023-05-31

**Authors:** Svenja Mehringer, Enrico Seiler, Felix Droop, Mitra Darvish, René Rahn, Martin Vingron, Knut Reinert

**Affiliations:** 1 Department of Mathematics and Computer Science, Freie Universität Berlin, Takustr. 9, 14195 Berlin, Germany; 2 MPI for Molecular Genetics, Ihnestr. 63, 14195 Berlin, Germany

**Keywords:** Approximate membership query, Sequence search, Miminizer, Alignment free analysis, Bloom filter, Metagenomics

## Abstract

We present a novel data structure for searching sequences in large databases: the Hierarchical Interleaved Bloom Filter (HIBF). It is extremely fast and space efficient, yet so general that it could serve as the underlying engine for many applications. We show that the HIBF is superior in build time, index size, and search time while achieving a comparable or better accuracy compared to other state-of-the-art tools. The HIBF builds an index up to 211 times faster, using up to 14 times less space, and can answer approximate membership queries faster by a factor of up to 129.

**Supplementary Information:**

The online version contains supplementary material available at 10.1186/s13059-023-02971-4.

## Background

Following the sequencing of the human genome [1, 2], genomic analysis has come a long way. The recent improvements of sequencing technologies, commonly subsumed under the term NGS (Next-Generation Sequencing) or 3rd (and 4th) generation sequencing, have triggered incredible innovative diagnosis and treatments in biomedicine, but also tremendously increased the sequencing throughput. Within 10 years, the current throughput of standard Illumina machines rose from 21 billion base pairs [1, 2] collected over months to about 3000 billion base pairs per day.

As a result of this development, the number of new data submissions, generated by various biotechnological protocols (ChIP-Seq, RNA-Seq, genome assembly, etc.), has grown dramatically and is expected to continue to increase faster than the cost per capacity of storage devices will decrease. This poses challenges for the existing sequence analysis pipelines. They are usually designed to run on a few recent samples, but cannot be used in reasonable time for thousands of samples, which makes it very costly to reanalyze existing data. Hence, we are collecting data that grows exponentially in size and are unable to reuse it in its entirety. Searching an entire database for relevant samples enables researchers to increase their sample size, a frequently problematic factor, and might even identify new relationships yet unknown. Limiting analysis to recently published data sets is a tremendous waste of resources and a large bottleneck for biomedical research.

The most basic task of such pipelines is to (approximately) search sequencing reads or short sequence patterns like genes in large reference data sets. This has led researchers to develop novel indexing data structures ([3], and shortly afterwards [4–7]) to search massive collections of sequences such as RNA-Seq files, pangenomes, or the bacterial and viral metagenome consisting of tens of thousands of species (see [8] for an overview). Recent tools tackling this problem (which we will compare to in this paper) are SeqOthello [9] (2018), Bifrost [7] (2019), COBS [10] (2019), Mantis [11] (2020), Raptor [12] (2021), and Metagraph [13].

SeqOthello is based on the Othello data structure and applies a *k*-mer-frequency-dependent storing scheme. Metagraph, Mantis, and Bifrost are based on de Bruijn graphs. While Bifrost additionally uses blocked Bloom filters, which are innately lossy, Mantis and Metagraph are exact methods. The former uses a counting quotient filter, and the latter stores a sequence index and annotation matrix. COBS and Raptor have a similar strategy, i.e., interleaving Bloom filters to gain efficient access to the *k*-mer occurrences per sample. Raptor uses the Interleaved Bloom Filter (IBF) as the main data structure. The IBF is a single, large bitvector taking advantage of the linearity regarding cache-access, whereas COBS stores a matrix of Bloom filters. COBS additionally applies a simple bin packing approach to reduce its index size.

In this work, we introduce a new data structure, the *Hierarchical Interleaved Bloom Filter* (HIBF) that overcomes major limitations of the IBF data structure. The HIBF successfully decouples the user input from the internal representation, enabling it to handle unbalanced size distributions and millions of samples. In contrast to COBS, which just groups samples into subindices, we compute a hierarchical layout that splits large samples, groups small ones, and distributes them into subindices based on their sequence similarity.

The results prove that we achieve a similar compression compared to COBS, but are orders of magnitudes faster when querying our index. In fact, the HIBF is faster than any method compared to in this work and has the smallest memory footprint in RAM while querying.

Moreover, the number of samples that can be used is essentially arbitrarily large. We exemplified this by scaling up to one million samples. This will enable many applications to distribute approximate searches onto very large data sets that occur in metagenomics, pangenomics, or sequence archives.

## Results

The data structures and tools mentioned in the introduction explicitly or implicitly address the following problem: Given a set of input sequences (samples), determine in which samples a query sequence can be found with up to a certain number of errors, also known as *Approximate Membership Queries* (AMQ). The most common approach is to store a representation of the samples’ sequence content in an index, which can answer whether a query belongs to one of the input sequences based on sequence similarity. To give an example, consider an input of 25,000 sets of bacterial species, where each set contains the sequences of genomes of all strains of the respective species. An index over this input can answer whether a query likely originates from one of the 25,000 species. In recent research, each set is called a *color* [5] or *bin, *[12]. We will use the term *bin*.

For efficiency, the sequence content is often transformed into a set of representative *k*-mers. The term *representative* indicates that the original *k*-mer content might be transformed by a function that changes its size and distribution, for example, by using winnowing minimizers [14] on the sequence and its reverse complement, or by using gapped *k*-mers [15]. A winnowing minimizer, which we call (*w*, *k*)*-minimizer*, is the lexicographically smallest *k*-mer of all *k*-mers and their reverse complements in a window of size *w*. When searching in an index, the same transformation is applied to the query.

### The HIBF

**Fig. 1:**
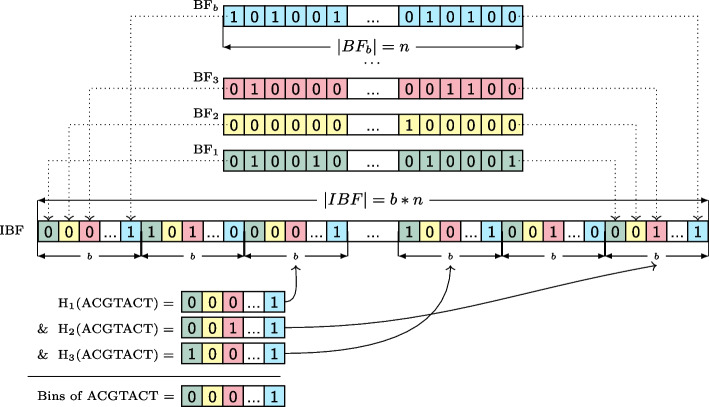
Example of an Interleaved Bloom Filter (IBF). Differently colored Bloom filters (BF) of length *n* for *b* bins (samples) are shown in the upper part. Interleaving the individual Bloom filters yields an IBF of size $b \times n$. In the example, we use three different hash functions to query a *k*-mer (ACGTACT) and retrieve 3 sub-bitvectors. By combining the sub-bitvectors with a bitwise &, we retrieve the *binning bitvector*, where a 1 indicates the presence of the *k*-mer in the respective bin

The Interleaved Bloom Filter (IBF) data structure published in [12] is a building block in the proposed Hierarchical Interleaved Bloom Filter (HIBF). Its general idea is depicted in Fig. 1, further details can be found in [12].

**Fig. 2:**
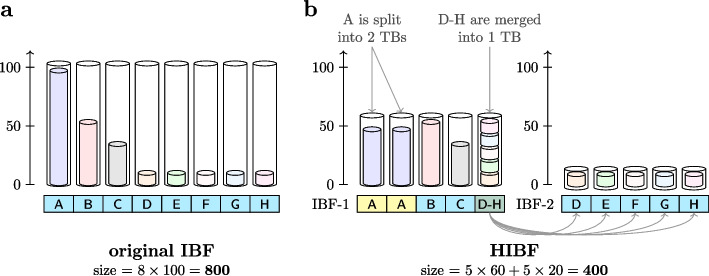
IBF vs. the HIBF. Given an input of eight *user bins* (UBs), subfigure **a** displays the layout of a normal IBF storing the content of the UBs, represented by the inner, lightly colored cylinders, in one *technical bin* (TB) each. The outer cylinders represent the size of the TBs and visualizes the wasted space. The horizontal rectangular bar represents the layout, indicating which UBs, identified by their ID (A-H), are stored in which TB. The same semantics hold for subfigure **b** which displays the corresponding HIBF with a maximum of 5 TBs on each level. In this example, UB A is split into the first two TBs in IBF-1, while UBs D-H are merged into the last TB. The *merged bin* requires a second lower-level IBF (IBF-2) storing the content of UB D-H in individual TBs. The size is given in exemplary numbers

The IBF data structure has two limitations. Firstly, due to the interleaved nature of the IBF, the individual Bloom filters must have the same size. Consequently, the largest Bloom filter determines the overall size of the IBF if we want to guarantee a maximal false-positive rate (Fig. 2a). Hence, for small-sized bins, the relatively large Bloom filters waste a lot of space. Secondly, although retrieving and combining sub-bitvectors is very efficient in practice, this only holds when the number of bins varies from a few hundreds to a few thousands. An increasing number of user bins will slow down the query speed.

The limitations of the IBF arise from the fact that the number of bins and their size directly determines its internal structure. To gain more independence from the input data, the Hierarchical Interleaved Bloom Filter (HIBF) explicitly distinguishes between user bins (UB) and technical bins (TB).

A *user bin* is equivalent to the former term *bin*, namely a set of sequences imbued with a semantic meaning by the user, e.g., all genomes of the species *Escherichia coli* could be in one user bin. Internally, the HIBF stores multiple IBFs on different *levels*. The bins of those IBFs are called *technical bins*. Technical bins may store parts of a user bin or contain the content of multiple user bins. In contrast, in the IBF, a user bin is also a technical bin, or simply a bin.

The main idea of the HIBF is to *split* large user bins into several technical bins and *merge* small user bins into a single technical bin to smooth the *k*-mer content distribution of the technical bins and thereby optimize the space consumption in an IBF (Fig. 2b). This distribution of user bins cannot be solved by a simple bin packing algorithm because of two reasons: (1) user bins can share *k*-mers, making the problem similar to the more complex *VM packing* [16] variant, and (2) splitting the content of user bins increases the complexity further and an applicable solution in the context of sequence analysis was not proposed as far as we know. Specifically, we will either:

1. *Split* the *k*-mer content of a user bin and store it in several technical bins
2. Store the entire *k*-mer content of a user bin in a *single* technical bin
3. Store the *k*-mer content of a range of user bins in one, *merged* technical bin

Splitting large user bins allows us to lower the maximum technical bin size, while merging small user bins avoids wasting space. However, we cannot use merged bins without further effort because when a query is contained in a *merged bin*, we cannot directly determine in which of the individual user bins the query is. For this purpose, we recursively add a rearranged IBF for each merged bin, with the corresponding merged user bins as input.

**Fig. 3:**
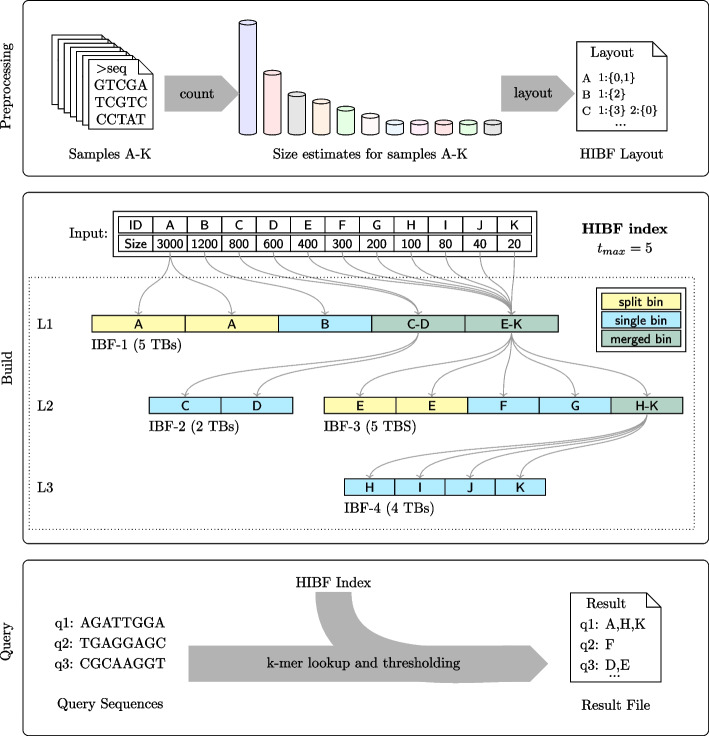
Workflow with details on the HIBF Index structure. Using the HIBF is done in three steps. (1) Preprocessing: Based on the size (representative *k*-mer content) of the input sequences, a layout is computed with the tool Chopper (see the “Availability of data and materials” section). (2) Build: From a given layout, Raptor (see the “Availability of data and materials” section) builds an HIBF index depicted in the middle box. (3) Query: The HIBF index can then be used to query the membership of sequences inside the input samples. The exemplary HIBF with $t_{max} = 5$ on 11 *user bins* (UB-A to UB-K) has 3 levels. The first level (L1) is always a single IBF (IBF-1) with exactly $t_{max}$ *technical bins* (TB). This IBF stores the full data set, structured in a way such that its size is significantly smaller than that of a normal IBF. The individual boxes inside an IBF represent its TBs, which store the *k*-mer content of the labeled *user bin(s)*. For example, in IBF-1 the content of UB-A is stored in two TBs (split), UB-B is stored in one TB (single), and UB-C to UB-D as well as UB-E to UB-K are collected in one TB each (merged). Subsequent levels may have several IBFs. Specifically, they will have one IBF for each *merged bin* on the previous level. For example, on the second level (L2), IBF-2 and IBF-3 correspond to the first and second merged bin of IBF-1, respectively. Note that the IBFs in the layout form a tree, where the root is the top-level IBF and the leaves are formed by IBFs without merged bins

The resulting collection of IBFs and their interconnectivity is what we call the Hierarchical Interleaved Bloom Filter, its detailed structure is stored in a *layout* (file). To compute a meaningful layout, we engineered a *dynamic programming* (DP) algorithm that optimizes the space consumption of the HIBF data structure given HyperLogLog size estimates of the input data (see the “ HyperLogLog estimates” section). From the layout, we can build the corresponding HIBF index, as exemplified in Fig. 3. Notably, every IBF in the HIBF except the top-level IBF stores redundant information. In the “Validation” section, we show that the space reduction on the top-level (Fig. 2 b IBF-1) usually compensates the extra space needed for lower levels.

Since the number of technical bins in each IBF of the HIBF is now independent of the number of user bins, we can choose a relatively small, fixed maximum number of technical bins $t_{max}$ which allows to efficiently query each IBF. We look up the *k*-mers of a query and apply a refined threshold on each level (see the “Querying an HIBF” section). Traversing lower levels is only required when a query is contained in a *merged bin*. In the “Validation” section we show that, on average, this seldom happens and that the positive impact of fixing the number of *technical bins* greatly outweighs the disadvantage of searching several levels.

### Validation

The Hierarchical Interleaved Bloom Filter (HIBF) data structure is incorporated in the tool *Raptor* [12] that can now be used with either the original IBF or the HIBF. We first show the improvement of the HIBF over the IBF on the simulated data set from the IBF paper [12]. Next, we compare against the existing tools on a metagenomic real-world data set consisting of 25,321 genomic sequences from the RefSeq database [17]. We compare ourselves against COBS, Mantis, Bifrost, Metagraph, SeqOthello, and the original IBF. HowDeSBT [18] could not handle this data set. In Additional file 1, we provide a third validation using a large RNA-Seq data set.

All benchmarks were performed on an Intel Xeon Gold 6248 CPU using 32 threads. We chose the parameters as proposed by the tutorials and help pages of all tools to the best of our knowledge, keeping equivalent parameters across tools consistent. The scripts can be found on our GitHub page^1^.

#### Simulated data

**Fig. 4:**
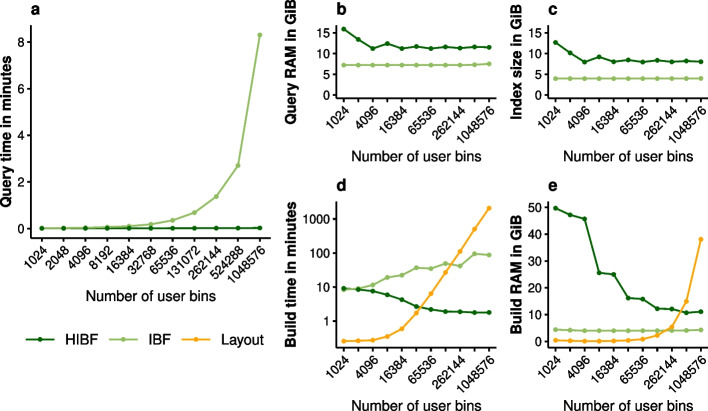
Simulated data set with increasing number of user bins. Each data point refers to one data set of 64 GiB divided into the respective number of user bins. This means that the total size of the data to index does not increase with an increasing number of user bins; the data is merely split into smaller parts. **a** shows the query time for 10 million reads, **b** the peak RAM usage during querying, **c** the total index size stored on disk, **d** the build time for constructing the index, and **e** the peak RAM usage during building

Following the approach in [12], we created a random DNA sequence of 4 GiB size and divided it into $b=[1024, 2048, \ldots , 1048576]$ *user bins* which would correspond to *b* different genomes, mimicking a metagenomic data set. Using the Mason genome variator [19], we then generated 16 similar genomes in each bin, which differ about $1\,\%$ on average. This could be seen as bins containing the genomes of homologous species. The total data set size is hence 64 GiB. Finally, we uniformly sampled ten million reads of length 250 bp from the genomes and introduced 2 errors in each read to simulate a sequencing experiment. This artificial data set is well-balanced, which is the ideal case for the IBF, since its overall size depends on the largest bin (Fig. 4).

We constructed the IBF and HIBF with canonical 32-mers and a false-positive rate of $5\,\%$. For the HIBF, $t_{max}$ was set to the square root of the number of user bins for each data set (see the “The choice of *t*_*max*_” section).

The results demonstrate that the HIBF successfully overcomes the limitations of the IBF during querying. While the IBF query time increases linearly with an increasing number of user bins, the HIBF is hardly affected. For a million user bins, the HIBF outperforms the IBF by two orders of magnitudes (297 times faster). The index size and, accordingly, the RAM usage during querying is higher for the HIBF on this data set. However, this is expected because the data set is perfectly balanced, and the hierarchical structure causes some overhead. The index construction time is slightly lower for the HIBF, even though the HIBF depends on the computationally expensive layout algorithm. Future versions of the layout computation will overcome this bottleneck.

#### Metagenomic data—RefSeq

This data set consists of 25,321 files containing the complete genomes of all Archaea and Bacteria in the RefSeq database [17] downloaded using the *genome_updater* (see the “Availability of data and materials” section) and has an uncompressed size of about 98.8 GiB. Compared to RNA-Seq data sets, which are often heavily filtered in a preprocessing step, the RefSeq sequences are non-repetitive and likely dissimilar. Thus, the input size is proportional to the *k*-mer content that needs to be indexed. Contrary to the simulated data set, this real-world data set is completely unbalanced. For example, the species *Escherichia coli*, represented by 634 assemblies, accounts for almost 7 % of all base pairs [20]. From the genomes, we simulated ten million queries. The number of queries per genome is proportional to its size, mimicking sequencing experiments. All tools were set up to compute canonical 32-mers, and we chose consistent parameters wherever possible. For the HIBF, $t_{max}$ was set to 192 (see “The choice of *t*_*max*_” section).

**Fig. 5:**
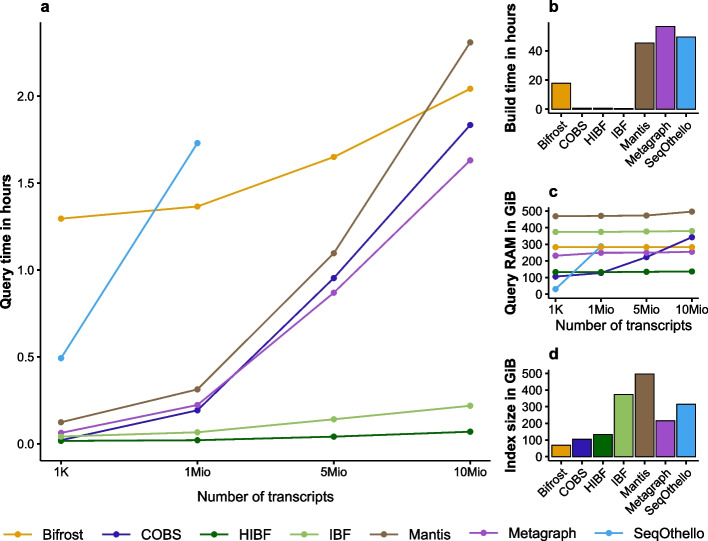
All complete genomes of Archaea and Bacteria in RefSeq. The uncompressed data set has a size of about 98.8 GiB. Ten million query reads of length $250\,bp$ were simulated using the *Mason simulator* [19]. The parameters used for all tools (if applicable) were: canonical *k*-mers $k=32$, no *k*-mer filtering, false-positive rate $5\,\%$, 2 hash functions, 32 threads, query search threshold 0.7. **a** Query time for varying number of transcripts, the best time of three runs was used. **b** Index build time, including all preprocessing steps. **c** Peak in RAM usage during querying for varying number of transcripts. **d** Index size stored on disk. Numeric values can be found in Additional file 1

For this data set, the HIBF outperforms all other tools in query time by several orders of magnitudes, and it has the smallest RAM footprint while doing so (see Fig. 5).

Additionally, the build time is significantly smaller than that of most other tools. The HIBF index is built in about 40 min, taking only slightly longer than the original IBF (32 min) and COBS (34 min). The other tools need several hours (Bifrost) or days (SeqOthello, Mantis, Metagraph).

The resulting index size is merely 133 GiB, which is only matched by COBS (104 GiB) and Bifrost (68 GiB). However, while having smaller indices when stored on disk, COBS and Bifrost have a much higher peak RAM usage while querying compared to the HIBF.

Most interestingly, regarding query times, the HIBF outperforms its closest contestant (Metagraph) by a factor of 24, requiring only 4 min for ten million queries. It improves on the original IBF by a factor of 3, while using a third of the RAM. The other tools need about $1.5-2$ h (Metagraph, COBS, Mantis, Bifrost). SeqOthello could not be run due to requiring more RAM than available on our system (1 TiB). The HIBF is the most efficient tool regarding the RAM usage while querying. It needs less than half the RAM of Metagraph, Bifrost, and COBS. COBS has a low RAM usage for few queries, but scales poorly with an increasing number of queries. While the same holds true for SeqOthello, all other tools have a steady RAM usage virtually independent of the number of queries.

Although not studied in other works so far, we would like to present the result file sizes of all tools. A small result file can speed up follow-up tasks in tool workflows. The (H)IBF result (10 million queries) is stored in merely 5 GiB, whereas COBS needs 18 GiB, Metagraph 95 GiB, Mantis 397 GiB, and Bifrost 473 GiB.

#### Flexible compression using minimizers

In our tool Raptor, we furthermore provide the possibility of compressing the input data using *minimizers*. When computing minimizers instead of *k*-mers, only the smallest (by some measure, e.g., lexicographically) *k*-mer in a window is stored. Thus, the number of *k*-mers is reduced while still representing the data accurately. We define (*w*, *k*)-minimizer as the set of all minimizers for a *k*-mer size *k* and a window length *w*.

**Fig. 6:**
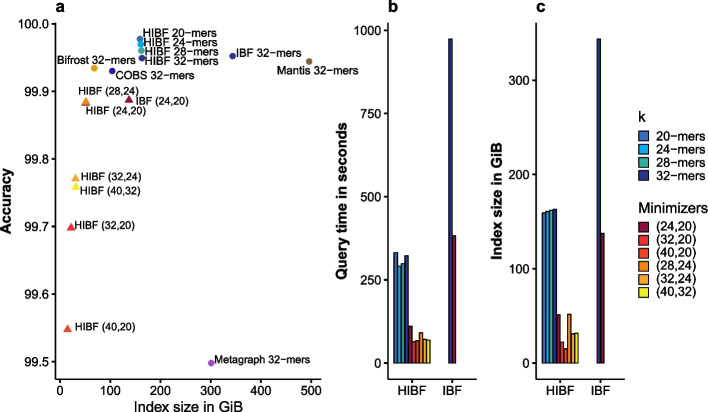
Additional experiments with varying *k* and (*w*, *k*) for the HIBF. The maximum false-positive rate for the (H)IBF was fixed to $1.5\%$, and we used $t_{max}=192$ for the HIBF. **a** For an HIBF using canonical *k*-mers, the choice of *k* had little impact on index size as well as accuracy and achieved the best overall accuracy. Compressing the index with minimizers decreased the accuracy. Although generally all tools achieve excellent accuracy, with a minimum of $99.5\%$ (Metagraph), it has to be noted that the accuracy is biased by a high number of true negatives. Thus, small differences in accuracy have a high impact on the actual numbers of false positives and negatives. **b** The index size in GiB. **c** The query time in seconds of the experiments on the RefSeq data set

We investigated the influence of varying values for *k* as well as (*w*, *k*) for the HIBF in terms of index size, query time, and accuracy (Fig. 6). Additionally, we included the accuracy of the other tools.

Choosing different values for *k* when using canonical *k*-mers did not significantly affect the index size, query time, or accuracy of the HIBF. In contrast, using minimizers could largely reduce the index size and query time.

A gentle compression with (24, 20)-minimizers already reduces the index size to a third of the 20-mer HIBF. A compression with (40, 32)-minimizer already reduces the size by a factor of 5 compared to a 32-mer HIBF. A small index using minimizers speeds up the query time (e.g., by a factor of 4 for (40, 32) minimizers) compared to the uncompressed HIBF. This further increases the advantage of the HIBF over other tools. The minimizer-compressed HIBF surpasses COBS, Mantis, and Bifrost by two orders of magnitudes (1 min vs. $\approx 2$ h).

Using the minimizer compression has a small negative impact on the accuracy. The larger the difference between *w* and *k*, the better is the compression, which in turn lowers the threshold for the query membership. This results in an increasing number of false positives, thus decreasing the accuracy. False negatives were rare and only slightly affected by the choice of (*w*, *k*). Other tools had a comparable accuracy to that of the HIBF using *k*-mers. None of the tools, except Mantis, had false negatives. While Mantis is an exact method for querying *k*-mers, its implementation makes it hard to apply the appropriate threshold for comparison with the HIBF, resulting in false-negative answers (see Additional file 1). The unexpected lower accuracy of Metagraph, which is an exact method, is subject to further investigation.

## Discussion

We presented the Hierarchical Interleaved Bloom Filter (HIBF), a novel data structure improving on its predecessor, the Interleaved Bloom Filter (IBF), by gaining flexibility over the input data, thereby optimizing the space consumption and markedly speeding up the query times. In our experiments, we could index 98.8 GiB of RefSeq data [17] (Archaea and Bacteria complete genomes as of 28-01-2022) in less than 13 min using 34.4 GiB of disk space. Querying all 25,321 samples for 10 million transcripts only took between 1 and 4 min. All other tools, i.e., COBS [10], Bifrost [7], Mantis [5], and Metagraph [13], need hours. Our RAM usage while querying the HIBF built with all *k*-mers was less than half of that of other tools. When using minimizer-compression, this memory footprint can be significantly lowered further, at the cost of a slightly lower accuracy. In summary, the HIBF enables indexing of large data sets on limited memory.

Moreover, the number of user bins (resp. colors) that can be used is virtually arbitrarily large. We exemplified this by using one million bins, improving over the query time of our original data structure by a factor of 300. While the layout computation in this scenario takes several hours, we are confident that we can devise special layout algorithms to speed up the layout computation and allow even more bins to be used with a reasonable layout time.

Since the build time of the HIBF is as low as that of COBS and about two orders of magnitude less than that of tools like Mantis, Bifrost, and Metagraph, the HIBF can be easily rebuilt even for very large data sets.

If we assume that a HIBF index needs about $40\%$ of the input size, and that we can handle millions of user bins, one can use the HIBF to index very large sequence repositories. For example, the European Nucleotide Archive (ENA) contains currently about 2.5 million assembled/annotated sequences with a total of $\approx 11$ Tbp [21]. Using the HIBF data structure, we could build 11 HIBF indices, each storing roughly 1 TiB of sequence content from about 250,000 *user bins*. We would expect each index to have a size of about 400 GiB, which easily fits into the main memory of a server (see Additional file 1, where we index 4 TiB of RNA-Seq files). We further showed that hundreds of thousands of *user bins* can be readily handled by the HIBF. Subsequently, querying ten million reads could be done by querying 11 HIBFs on different machines in parallel in less than 2 min. A handful of queries would be answered in a fraction of a second. Hence, in this setting, the archives could offer a portal for individual user queries, knowing that they can answer about 100,000 queries (of length 250) per second.

## Conclusion

In this work, we presented a general data structure and method for storing a representative set of *k*-mers of a data set that is partitioned into hundreds up to millions of user bins in such a way that very fast approximate membership queries are possible. Large sequence databases could now be indexed in a fraction of their original size and support sequence queries in a couple of minutes. The HIBF data structure has enormous potential. It can be used on its own, like in the tool Raptor, or can serve as a prefilter to distribute more advanced analyses such as read mapping.

## Methods

The following sections provide the details referred to in the “Results” section. We will use the following notation:

$$\varvec{k}$$
*k*-mer size
$$\varvec{w}$$
window size for minimizers
$$\varvec{h}$$
number of hash functions
$$\varvec{m}$$
size (in bits) of an individual Bloom filter
$$\varvec{n}$$
number of values (*k*-mers) stored in an individual Bloom filter
$$\varvec{p}_{\varvec{fpr}}$$
false-positive rate
$$\varvec{b}$$
number of (*user*) bins indexed in an (H)IBF
$$\varvec{t}$$
number of (*technical*) bins of the IBF data structure
$$\varvec{t}_{\varvec{max}}$$
maximum number of technical bins in each IBF of the HIBF
$$\varvec{c(w,k)}$$
expected decrease in the amount of representative *k*-mers when switching from *k*, *k*-minimizers to *w*, *k*-minimizers
$$\varvec{x}$$
number of minimizers in a query
$$\varvec{a}$$
number of false-positive answers
$$\varvec{b}_{\varvec{p}}\varvec{(x,a)}$$
probability of returning *a* false-positive answers when querying *x* minimizers in a Bloom filter of a false-positive rate $p_{fpr}$
$$\varvec{t(x)}$$
threshold for a given *x*
$$\varvec{c}$$
(constant) correction that is added to the threshold
$$\varvec{p}_{\varvec{max}}$$
threshold for correction term; i.e., increase *c* until $b_p(x, a+c) < p_{max}$

### The HIBF

#### Computing an HIBF Layout—a DP algorithm

In this section, we explain how to compute a layout, i.e., how we decide which user bins to split and which to merge (Fig. 3). To this end, we engineered a dynamic programming (DP) algorithm that heuristically optimizes the space consumption of a single IBF, while considering the space needed for lower level IBFs for *merged bins*. Thus, computing the layout on the first, main level IBF optimizes its layout while estimating the size of the entire HIBF. As outlined in “The HIBF” section, we then recursively apply the DP algorithm on each lower level IBF.

Assume that we have *b* user bins $\text {UB}_0,\ldots ,\text {UB}_{b-1}$ sorted in decreasing order of their size, $|\text {UB}_0| \ge \ldots \ge |\text {UB}_{b-1}|$, which we want to distribute across *t* technical bins $\text {TB}_0,\ldots , \text {TB}_{t-1}$ in a single IBF. While not strictly necessary, the *order* is chosen such that small user bins cluster next to each other because in our algorithm only contiguous bins may be merged. We denote the *union* size estimate of a *merged bin* that stores the *k*-mer multiset $\text {UB}_i \cup \text {UB}_{i+1} \cup \ldots \cup \text {UB}_j$ as $U_{i,j}$. We use HyperLogLog sketches [22] (see the “Union estimation” section) to quickly estimate the size of the union when merging user bins. Since the IBF only stores a representative *k*-mer’s presence (*k*-mer content), not how often it was inserted, the size of the *merged bin* may be smaller than the sum of sizes of the respective user bins. The effect is that merging user bins of similar sequence content results in smaller sized *merged bins* which is beneficial for the overall size of the IBF. We exploit this advantage by the optional step of rearranging the user bins based on their sequence similarity (see the “Rearrangement of user bins” section).

Further, the user needs to fix the number of hash functions *h*, the desired false-positive rate $p_{fpr}$, and the maximum number $t_{max}$ of technical bins to be used for each IBF of the HIBF. The former two parameters are needed to estimate the IBF size and must correspond to the parameters of building the index afterwards. The latter is critical for the HIBF layout. See the “Recommended choice of parameters” section for a discussion of sensible defaults.

Regarding the expected false-positive rate of the HIBF index, we point out that when splitting a user bin, we introduce a *multiple testing problem*. This happens because we query a *k*-mer for a split bin several times in several technical bins. We correct for this by increasing the size of the respective technical bins by a factor $f_{corr}(s, p_{fpr})$ where *s* is the number of technical bins the user bin is split into and $p_{fpr}$ is the desired false-positive rate (see the “IBF size correction for split bins” section).

The *general sketch* of the algorithm is the following: The dynamic programming (DP) matrix *M* has *t* rows, representing $\text {TB}_0,\ldots , \text {TB}_{t-1}$ and *b* columns, representing $\text {UB}_0,\ldots , \text {UB}_{b-1}$. When we move horizontally in the matrix, we consume multiple user bins while remaining in a single technical bin. This indicates a merged bin. When we move vertically, we consume multiple technical bins while remaining in a single user bin. This indicates a split bin. We treat a *single bin*, introduced for clarity in the “Methods” section, as a split bin of size 1. We do not move along the diagonal. The above semantics allow us to verbalize a structure that is then used to estimate the space consumption and compute a local optimum in each cell of the DP matrix. Specifically, the space consumption is tracked using two $t \times b$ matrices $M_{i,j}$ and $L_{i,j}$:

- $M_{i,j}$ tracks the maximum technical bin size $\underset{g \in {0,...,i}}{\max }(|\text {TB}_g|)$ when the first $j + 1$ user bins are distributed to $i + 1$ technical bins. Minimizing the maximum technical bin size optimizes the IBF space consumption, since it directly correlates with the total IBF size (Fig. 2).
- $L_{i,j}$ tracks the space consumption estimate of all lower level IBFs that need to be created for each merged bin given the structure imposed by $M_{i,j}$.

##### Initialization

For the first column, when $j = 0$, there is only a single user bin $\text {UB}_{0}$ which can be split into *t* technical bins. Therefore, the maximum technical bin size stored in $M_{i,0}$ is the size of $\text {UB}_{0}$ divided by the current number of technical bins *i* it is split into, corrected by $f_{corr}$ (see the “IBF size correction for split bins” section). Since no merging is done, no lower level IBFs are needed and $L_{i,0}$ is always 0.

1 $$\begin{aligned} \forall i \in \{0, \ldots , t-1\}&:&M_{i, 0}=\frac{|\text {UB}_{0}|}{i+1}\cdot f_{corr}(i+1,p_{fpr})\end{aligned}$$

2 $$\begin{aligned} \forall i \in \{0, \ldots , t-1\}&:&L_{i, 0}=0 \end{aligned}$$

For the first row, when $i = 0$ and $j\ne 0$, all user bins have to be merged into a single technical bin. The size of the resulting *merged bin* is estimated using the precomputed unions $U_{0,j}$ (see the “Union estimation” section). Since we create a *merged bin* in each step, the resulting additional space consumption on lower level IBFs is estimated by the sum of sizes of contained user bins times the maximal number of levels $l=\lceil \log _{t_{max}}(j+1)\rceil$. We use the sum instead of the union here because we do not yet know how the lower level IBFs are laid out, so we estimate the worst case of storing every *k*-mer again on each lower level.

3 $$\begin{aligned} \forall j \in \{1, \ldots , b-1\}&:&M_{0, j}=U_{0,j}\end{aligned}$$

4 $$\begin{aligned} \forall j \in \{1, \ldots , b-1\}&:&L_{0, j}= l \cdot \sum _{g=0}^j |\text {UB}_g| \end{aligned}$$

**Fig. 7:**
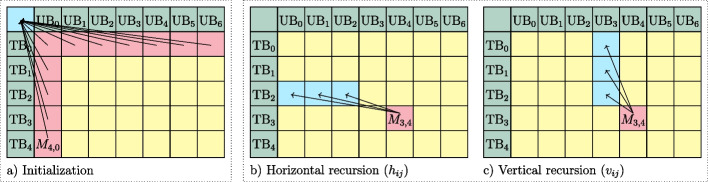
DP algorithm traceback visualization. Matrices **a**, **b**, and **c** visualize the algorithm for a layout distributing $b=7$ user bins (columns) to $t=5$ technical bins (rows). **a** Shows the traceback of the initialization, all coming from the virtual starting point $(-1,-1)$. E.g., $M_{4,0}$ represents the sub-layout of splitting $\text {UB}_0$ into all available technical bins. **b** Shows which cells $j' < j - 1 = 3$ are considered in the horizontal recursion. E.g., $j' = 2$ would indicate to merge $\text {UB}_3$ and $\text {UB}_4$ into $\text {TB}_3$. **c** Shows which cells $i' < i = 3$ are considered in the vertical recursion. E.g., $i' = 1$ would indicate to split $\text {UB}_4$ into $\text {TB}_2$ and $\text {TB}_3$

##### Recursion

We store the maximum technical bin size in $M_{i,j}$ and the lower level costs in $L_{i,j}$ and we want to optimize the total HIBF space consumption. It is computed by $M_{i,j}$ times the number of technical bins, which is the size of the first, main level IBF, plus the lower level costs $L_{i,j}$:

5 $$\begin{aligned} |HIBF| = M_{i,j} * (i + 1) + \alpha * L_{i,j} \end{aligned}$$

The *parameter* $\alpha$ can be used to tweak the influence of lower levels on the space and query time of the HIBF. The query time increases when many lower levels are introduced since we have to traverse and query a lot of IBFs, but the space consumption is often lower. When $\alpha = 1$, the space consumption is optimized by expecting our lower level space estimate to be exact. If we choose higher values for $\alpha$, we artificially increase the costs of *merged bins* and their lower levels, thereby decreasing their occurrence in the layout. A sensible default that was experimentally derived to work well is $\alpha = 1.2$.

In the recursion, we process each cell $M_{i,j}$, $i \ne 0$ and $j\ne 0$, by deciding for the next user bin $\text {UB}_j$ whether to (1) split it into $i - i'$, for some $i' < i$, technical bins, or (2) merge it with all user bins starting at $j'$, for some $j' < j$.

In the first case, when *splitting* $\text {UB}_{j}$ and thereby moving vertically, we want to find the $i' < i$ that results in the smallest overall HIBF size $v_{i,j}$ (Fig. 7 c). Semantically, we originate from the layout $M_{i',j-1}$ which already distributed $\text {UB}_0,\ldots ,\text {UB}_{j-1}$ into $\text {TB}_0,\ldots ,\text {TB}_{i'}$, leaving the technical bins $\text {TB}_{i' + 1},\ldots ,\text {TB}_{i}$ to store the split content of $\text {UB}_{j}$. The new HIBF size is computed analogous to Eq. 5 with the new maximal technical bin size $m_v$ and the new lower level costs $l_v$. Since we introduce no *merged bin*, $l_v$ is simply the former costs $L_{i',j-1}$. $m_v$ is computed by taking the maximum of the former value $M_{i',j-1}$ and the *split bin* size we obtain from equally dividing the *k*-mer content of $\text {UB}_{j}$ into $i - i'$ technical bins corrected by $f_{corr}$.

6 $$\begin{aligned} v_{i, j}=\min _{i' \in \{0, \ldots , i-1\}}\left( \underbrace{\max \left( M_{i', j-1}, \frac{|\text {UB}_{j}|\cdot f_{corr}(i-i', p_{fpr}) }{i-i'}\right) }_{m_v} \cdot (i+1)+\alpha \cdot \underbrace{L_{i', j-1}}_{l_v}\right) \end{aligned}$$

In the second case, when *merging* $\text {UB}_{j}$ and thereby moving horizontally, we want to find the $j' < j - 1$ that results in the smallest overall HIBF size $h_{i,j}$ (Fig. 7 b). Semantically, we originate from the layout $M_{i-1,j'}$ which already distributed $\text {UB}_0,\ldots ,\text {UB}_{j'}$ into $\text {TB}_0,\ldots ,\text {TB}_{i-1}$, leaving technical bin $\text {TB}_{i}$ to store the merged content of $\text {UB}_{j'+1},\ldots ,\text {UB}_{j}$. The new HIBF size is computed analogous to Eq. 5 with the new maximal technical bin size $m_h$ and the new lower level costs $l_h$. $m_h$ is computed by taking the maximum of the former value $M_{i-1,j'}$ and the *merged bin* size we obtain from the precomputed Union $U_{j'+1,j}$. Since we introduce a new *merged bin*, $l_h$ is computed by adding to the former costs $L_{i-1, j'}$, the space estimate of *l* expected lower levels times the sum of contained user bin sizes (see Eq. 4).

7 $$\begin{aligned} h_{i,j}=\min _{j' \in \{0, \ldots , j-2\}}\left( \underbrace{\max \left( M_{i-1, j'}, U_{j'+1,j} \right) }_{m_h} \cdot (i+1)+\alpha \cdot \underbrace{\left( L_{i-1, j'}+l\cdot \sum _{g=j'+1}^j |\text {UB}_g|\right) }_{l_h}\right) \end{aligned}$$

Given $m_v$, $l_v$, $m_h$, and $l_h$ for which the minima in $v_{i,j}$ and $h_{i,j}$ are achieved, respectively, the values of the cells $M_{i, j}$ and $L_{i, j}$ are updated according to the minimum of $v_{i,j}$ and $h_{i,j}$:

8 $$\begin{aligned} M_{i, j}=\left\{ \begin{array}{ll} m_{v} &{} \text{ if } v_{i, j} \le h_{i, j} \\ m_{h} &{} \text{ else } \end{array} \quad L_{i, j}=\left\{ \begin{array}{ll} l_{v} &{} \text{ if } v_{i, j} \le h_{i, j} \\ l_{h} &{} \text{ else } \end{array}\right. \right. \end{aligned}$$

The above recurrence can be used to fill the two dynamic programming matrices row- or column-wise because the value of a cell depends entirely on cells with strictly smaller indices. The traceback can then be started from the cell $M_{t-1,b-1}$.

Since we use HyperLogLog sketches, the algorithm runs quite fast in practice. For example, it takes only 13 min to compute the layout with $t_{max}=192$ of user bins for a data set consisting of all complete genomes of archaea and bacteria species in the NCBI RefSeq database [17] (about 25,000 genomes with total size of roughly 100 GiB).

#### HyperLogLog estimates

The *HyperLogLog* (HLL) algorithm [22] approximates the number of distinct elements in the input data that were previously converted into a set of uniformly distributed, fixed-size hash values. It is based on the observation that a hash value of length $q>p$ has a probability of $\frac{1}{2}^{p}$ of containing *p* leading zeros. When keeping track of the maximum number of leading zeros *lz* of the data’s hash values, one can estimate its size via $2^{lz}$. Since this estimate has a very high variance, it is improved by splitting the data into $m = 2^b$, $b \in [4, q-1]$, subsets, and keeping track of *lz* for each subset. These *m* values are called a *sketch* of the input data. The size estimate of the original input is computed by using the harmonic mean and bias correction of the *m* values of the subsets (see [22] for details).

In our application, we convert the input sequences into 64-bit *k*-mers by arithmetic coding, hash them using the third-party function XXH3_64bits^2^, and then apply the HLL algorithm with $q=64$ and $b=12$ using our adjusted implementation of the *HyperLoglog* library^3^.

#### Union estimation

Given two *HyperLogLog* sketches, e.g., created from two user bins, with the same number of values *m*, they can be merged by inspecting the *m* values of both sketches and simply storing the larger value (*lz*) of each. The resulting new sketch can estimate the size of the combined sketches, namely the number of distinct elements in the union of the two user bins. When merging several *sketches*, we merge the first two and then merge the rest iteratively into the new union sketch.

In our application, when iterating column-wise over the matrix of the DP algorithm, we precompute for each column *j* the $j-1$ union estimates $U_{j',j}$ for $j' \in [0, ... , j-1]$ with

9 $$\begin{aligned} U_{i,j} = \text {UB}_i \cup \text {UB}_{i+1} \cup \ldots \cup \text {UB}_j. \end{aligned}$$

#### Rearrangement of user bins

When merging two or more user bins, we unite their *k*-mer content by storing all unique *k*-mers present in each user bin. It follows that the size of the union is always smaller or equal to the sum of sizes of the united user bins. For a merged bin in the HIBF, this means that we can potentially save space by uniting user bins of similar *k*-mer content. To exploit this advantage, the user has the option to partially rearrange the sorted order of user bins based on their similarity.

We estimate the similarity of two user bins by computing the *Jaccard distance* based on *HyperLogLog* sketches. The *Jaccard distance* $d_J(A,B)$ is defined as 1 minus the *Jaccard index* *J*(*A*, *B*) and can be rewritten in the following way:

10 $$\begin{aligned} {d_{J}(A,B)=1-J(A,B)=2- \frac{|A|+|B|}{|A\cup B|}} \end{aligned}$$

We use the rightmost term to compute the *Jaccard distance* using the union sketch of A and B to estimate $|A\cup B|$.

**Fig. 8:**
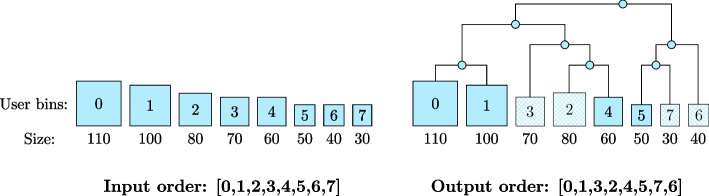
Rearranging user bins by agglomerative clustering. Given eight user bins (UBs) as input, sorted by their size (left-hand side), the example shows how the order may change after applying an agglomerative clustering algorithm based on sequence similarity (right-hand side). The agglomerative clustering arranged UB-2 and UB-4 next to each other, as well as UB-5 and UB-7

When rearranging, we still want to partly maintain the initial order of user bin sizes for two reasons: (1) for the HIBF it is beneficial to merge small user bins (see Fig. 2), and (2) a limitation of the DP algorithm is that only user bins next to each other can be merged, so ordering them by size clusters small bins together. We therefore only rearrange user bins in non-overlapping *intervals*. The size of an interval is determined by the parameter $r_m$, the maximum ratio of the largest to the smallest user bin within the interval.

Within an interval, we perform an agglomerative clustering on the range of user bins based on their *Jaccard distances*. The order of user bins is changed according to the resulting cluster tree, leaving similar user bins adjacent to each other (see Fig. 8).

#### IBF size correction for split bins

Recall that for a given false-positive rate $p_{fpr}$, the size in bits *m* of an individual Bloom filter is proportional to the amount of *k*-mers *n* it is designed to store. If *h* is the number of hash functions, then it holds:

11 $$\begin{aligned} m \approx -\frac{h\cdot n}{\ln (1 - p_{fpr}^\frac{1}{h})} \end{aligned}$$

If we split a user bin into *s* technical bins and divide the *k*-mer content equally among them, we cannot simply use the above equation 11 on each technical bin to determine its size because we introduce a multiple testing problem. Specifically, the probability for obtaining a false-positive answer for the user bin when querying those *s* technical bins is $1-(1-p_{fpr})^{s}$, since we only get no false-positive answer if *all* *split bins* have no false-positive answer. We can determine the required false-positive rate $p_{corr}$ such that conducting multiple testing yields the desired false-positive rate $p_{fpr}$:

12 $$\begin{aligned} p_{corr} = 1 - (1 - p_{fpr})^\frac{1}{s} \end{aligned}$$

We use $p_{corr}$ to determine a factor for increasing the size of *split bins* to mitigate the effect of multiple testing:

13 $$\begin{aligned} f_{corr}(s,p_{fpr})=\frac{\ln (1-p_{fpr}^\frac{1}{h})}{\ln (1-p_{corr}^\frac{1}{h})} \end{aligned}$$

More details can be found in Additional file 1. For example, given $h=4$ and $p_{fpr}=0.01$, if we split a user bin into $s=5$ technical bins, their size computed with equation 11 must be increased by the factor 1.598. If we split the same user bin into $s=20$ technical bins, the size must be corrected by 2.344.

#### Building an HIBF index from a layout

The hierarchy in an HIBF layout forms a tree (Fig. 3). To avoid reading input files several times, we build the index using a *bottom-up* strategy implemented by a recursive construction algorithm. The recursion anchor lies at IBFs without merged bins (leaves of the tree) and traverses the layout similar to a *breadth-first search*. At a leaf, we read the respective input samples and transform them into their representative *k*-mer content. Based on the *k*-mer contents, we can construct an IBF whose size fits the desired maximum false-positive rate. We then insert the *k*-mer contents into the IBF. Moving along the tree, we keep the *k*-mer contents of child nodes to insert them into the respective merged bins of parent IBFs. At a parent node, the input samples from split and single bins are read, processed and, together with the merged bin content from child nodes, used to construct the current IBF. The algorithm ends at the root IBF on level L1. We trivially parallelized the construction of independent lower levels.

#### Querying an HIBF

To query the HIBF for a sequence, we employ a *top-down* strategy. First, the query sequence is transformed into representative *k*-mers in the same way as it was done for the index construction. For each *k*-mer, we determine its membership in the first, main level IBF of the HIBF by counting the total number of *k*-mers that occur in each *technical bin*. Subsequently, we gather all *technical bins* whose count is equal to or exceeds a certain *threshold*. For *split bins*, the *k*-mer counts of all bins associated with the respective *user bin* need to be accumulated before thresholding. For *split* and *single bins*, we can directly obtain the corresponding *user bins* to answer the query. For *merged bins* that exceed the threshold, we need to apply the same procedure recursively on the associated child IBF on a lower level. Notably, having a threshold allows us to skip traversal of lower level IBFs whose upper level *merged bins* do not exceed the threshold. In practice, this means that we only have to access a fraction of the HIBF. The final result is the set of all *user bins* that contain a query.

The threshold can be either user-defined or computed for a given amount of allowed errors *e* in the query. For the latter, we rely on the method introduced in [12]. Briefly, we utilize a well-known *k*-mer lemma [23]. However, when winnowing minimizers are used, we apply a probabilistic model that accounts for how errors might destroy relevant *k*-mers [12]. The model returns a threshold value given how many representative *k*-mers are in the query. In this work, we further refined the model to incorporate the effect of the false-positive rate on the threshold (see the “Impact of the HIBF’s false-positive rate” section). The thresholding step is a convenience for the user missing in tools like Mantis [5].

### Validation

#### Key parameters

The HIBF has many parameters that influence the memory consumption and run time, and partially depend on each other. We acknowledge that this can be challenging for the user. To alleviate the problem, we will in the following sections (1) describe the impact of the key parameters on the performance of the HIBF and (2) give sensible defaults and describe how the HIBF can set them automatically given very few, easy to understand parameters.

##### The choice of *t*_*max*_

The choice of $t_{max}$, i.e., the maximum number of technical bins of the individual IBFs in an HIBF, influences the depth of the HIBF which impacts both total index size and query runtime. We show in the following that the optimal $t_{max}$ is near the square root of the number of user bins *b*, i.e. $sq=\lceil \sqrt{b}/64\rceil \cdot 64$.

If $t_{max}$ *is relatively small*, the depth of the HIBF is large, but the individual IBFs will be small. In terms of query runtime, querying a small IBF is fast, but we potentially have to traverse many levels. In terms of space, a small $t_{max}$ results in storing a lot of redundant information and thus the space consumption increases. If we choose $t_{max}$ *relatively large*, the depth of the HIBF and the number of IBFs will be low, but the individual IBF sizes will be large. In terms of query runtime, we then expect few queries on each level, but querying an IBF is more costly. In terms of space, the larger $t_{max}$, the more technical bins we have to cleverly distribute the content of the user bins to and thus lower the space consumption. For some values of $t_{max}$, the space consumption increases, although $t_{max}$ is large. This happens because IBFs on the last level contain only a few UBs, which will lead to relatively high correction factors $f_h$ (see the “IBF size correction for split bins” section).

**Table 1:** Runtime penalty for an increasing number of bins in an IBF. The values represent ratios by which the query run time increases when using an IBF with *b* bins compared to an IBF with 64 bins. We simulated *b* equally sized (*user*) bins for $b\in \{64,256,512,...,32768\}$ of random sequence content (1 GiB in total) and sampled 1  million reads of length 250. We then constructed an IBF over these *b* bins using 32-mers and 2 hash functions. We conducted this experiment for different false-positive rates, including 0.0001, 0.0125, and 0.3125, which resulted in IBFs of different densities. Next, we measured the time required to count the *k*-mer occurrences in each of the *b* bins for all 1 million reads. The reported values are the average of five repetitions

$\textbf{b}$	64	128	256	512	1024	2048	4096	8192	16384	32768
$p_{fpr}=0.0001$	1.00	1.06	1.35	1.35	1.57	1.96	3.41	5.49	6.81	10.95
$p_{fpr}=0.0125$	1.00	1.01	1.17	1.34	1.83	2.70	5.39	8.62	15.05	28.33
$p_{fpr}=0.3125$	1.00	1.28	1.55	2.26	3.78	6.54	12.94	24.45	47.63	93.47

With a simple experiment^4^, we validated one of the above observations, namely that the query runtime of a single IBF increases with the number of (technical) bins (see Table 1). The results show an increase in query runtime for each false-positive rate. Additionally, we observe that the higher the false-positive rate, the greater the query runtime penalty with an increasing number of bins. We can use those results to optimize the choice of $t_{max}$.

**Fig. 9:**
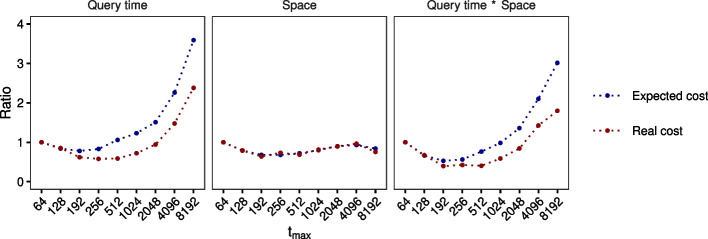
Expected cost versus real cost. The data are all complete genomes of archaea and bacteria from the RefSeq database. The relative expected cost was computed with our model, while the relative real cost was measured with a $t_{max}$-HIBF with $p_{fpr}=0.015$, 4 hash functions and (24, 20)-minimizer. The cost is given as a ratio of expected cost to real cost for a 64-HIBF because measurements are platform-dependent. The correlation is $> 0.9$, although we systematically overestimate the required time. Nevertheless, the three best $t_{max}$ are in both cases 192, 256, and 512 with predicted values 0.53, 0.56, and 0.75 and real values 0.39, 0.42, and 0.40

To investigate advantageous values of $t_{max}$ for a given data set, the user of our tool has the possibility to *compute statistics* on their input data. They have to fix the following three parameters beforehand: (1) the *k*-mer size, (2) the number of hash functions, and (3) the maximum false-positive rate (see the “Recommended choice of parameters” section). The statistics then give an estimation on the *expected cost* of the index size and query time of an HIBF for several values of $t_{max}$. As an example, we computed the statistics for the real-world data of the RefSeq database from our experiments and validated them by actually building and querying a respective HIBF (Fig. 9). The results show an excellent correlation between our prediction and the actual product of run times and memory consumption, namely a correlation of 0.95 for $t_{max}$ ranging from 64 to 8192. We were able to estimate the index size very closely to the real size, while we slightly underestimated the query time for increasing values of $t_{max}$. In the combination of space and query time, we identified $t_{max} = 192$ as the optimal choice in both the expected total cost and real total cost, where 192 is a multiple of the word size 64 that is near the square root of the number of user bins *b*, i.e., $sq=\lceil \sqrt{b}/64\rceil \cdot 64$. In general, our tool computes the statistics for $t_{max}=sq,64,128,256,\ldots$ until the product $\textit{query time} \cdot \textit{space}$ increases. For the above example (Fig. 9), we would stop at $t_{max}=256$ since the product increases and choose $t_{max}=192$ as the optimal value.

In summary, given input data, we can compute a $t_{max}$ that minimizes the expected query time of the HIBF or the minimal expected run time weighted with memory consumption and free the user from choosing the parameter as long as the data in the UBs is not similar. We postulate that this strategy works well if a query is on average only contained in one (or a few) UBs.

##### Choice of *w* and *k*

To lower the space consumption and accelerate queries, we support (*w*, *k*)-minimizers. Other methods for shrinking the set of representative *k*-mers like described in [24] or [25, 26] are also possible for the HIBF. For a detailed description of (*w*, *k*)-minimizers, see [12]. The authors showed that, for up to 2 errors, parameters $w=23$ and $k=19$ perform well. In general, the larger we make *w* compared to *k*, the fewer representative *k*-mers we have to store. However, the accuracy decreases, and we might increase the number of false positives and false negatives. In comparison, Kraken2 [27] uses values of $w=35$ and $k=31$.

A *k*-mer is identified as a minimizer if it has the smallest value in any of the windows of length *w* which cover the *k*-mer. Following the argument of Edgar [26], we can define the compression factor *c*(*w*, *k*) to be the number of *k*-mers in a window divided by the number of minimizers. Hence, $c(w,k)\ge 1$ and larger *c*(*w*, *k*) indicates a smaller set of representative *k*-mers. For (*w*, *k*)-minimizers, *c*(*w*, *k*) can be estimated as follows: Consider a pair of adjacent windows of length *w* in a random sequence. Sliding one position to the right, two *k*-mers are discarded from the first window (the *k*-mer and its reverse complement) and two are added to the second. The minimizer in the second window is different from the one in the first window if one of the four affected minimizers has the smallest value over both windows; otherwise, the minimizer of both windows is found in their intersection and does not change. There are $2\cdot (w-k+2)$ *k*-mers in the two windows combined, and the probability that a given one of these has the smallest value is $p = 1/(2\cdot (w-k+2))$. Thus, the probability that a new minimizer is introduced by sliding the window one position is $4\cdot p = 2/(w -k + 2)$ and hence

$$\begin{aligned} c(w,k)\approx \frac{w-k+2}{2} \end{aligned}$$

For (23, 19)-minimizers, we would expect a compression by a factor of 3. For (32, 18)-minimizers, we would expect a compression factor of 8. Using a larger *w* is beneficial for saving space. On the other hand, the threshold for determining a match becomes smaller (roughly by the compression factor, for details see [12]). We want to avoid having a threshold of only 1 or 2, since a few false-positive *k*-mer matches would then result in a false-positive answer.

##### Impact of the HIBF’s false-positive rate

Recall that we ensure that the overall false-positive rate for querying an HIBF does not exceed a certain threshold. Note that, for a given false-positive rate $p_{fpr}$, the size in bits *m* of an individual Bloom filter is proportional to the amount of *k*-mers *n* it is designed to store. If *h* is the number of hash functions, then it holds:

$$\begin{aligned} m \approx -\frac{h\cdot n}{\ln (1 - p^\frac{1}{h})} \end{aligned}$$

Since we aim at making each IBF in the HIBF as small as possible, we will have to accommodate a relatively high false-positive rate.

We use the counts of the (*w*, *k*)-minimizers and the probabilistic threshold derived in [12] to decide whether a query is in a bin. However, having a relatively high false-positive rate $p_{fpr}$ will affect this thresholding. If a query has *x* minimizers, we can compute the probability that *a* of them return a false-positive answer in an IBF with false-positive rate $p=p_{fpr}$ as

$$\begin{aligned} b_p(x,a)=\left( {\begin{array}{c}x\\ a\end{array}}\right) \cdot p^a \cdot (1-p)^{(x-a)} \end{aligned}$$

This can be quite significant. For example, for queries of length 250, we have the distribution of minimizers (computed for 1 million random reads) depicted in columns 1 and 2 of Table 2.

**Table 2:** Exemplary threshold distribution. The values are for (38, 20)-minimizers, 2 errors, and 1 million reads of length 250. Shown are the distribution $\#x$ ($\%x$) of the number (percentage) of minimizers reaching from $x=14$ to $x=35$ and the threshold *t*(*x*) using the probabilistic model from [12]. The threshold *t*(*x*) incorporates the correction term $c_p$. On the right, the probability $b_p(x,a)$ of having *a* false-positive answers from the IBF with $p=0.05$ is shown

*x*	#*x*	%*x*	*t*(*x*)	$c_{0.05}$	$b_{0.05}(x,1)$	$b_{0.05}(x,2)$	$b_{0.05}(x,3)$
14	6	<0.1	5	1	35.9	*12.3*	2.6
15	214	<0.1	6	1	36.6	*13.5*	3.1
16	2059	0.2	6	1	37.1	*14.6*	3.6
17	11,081	1.1	8	2	37.4	15.8	*4.1*
18	36,651	3.5	9	2	37.6	16.8	*4.7*
19	83,748	8.0	9	2	37.7	17.9	*5.3*
20	139,864	13.3	10	2	37.7	18.9	*6.0*
21	179,962	17.2	11	2	37.6	19.8	*6.6*
22	185,842	17.7	12	2	37.5	20.7	*7.3*
23	158,032	15.1	12	2	37.2	21.5	*7.9*
24	113,696	10.8	13	2	36.9	22.3	*8.6*
25	70,089	6.7	14	2	36.5	23.1	*9.3*
26	37,540	3.6	15	2	36.1	23.7	*10.0*
27	18,040	1.7	15	2	35.6	24.3	*10.7*
28	7535	0.7	16	2	35.0	24.9	*11.4*
29	2790	0.3	17	2	34.5	25.4	*12.0*
30	961	<0.1	18	2	33.9	25.9	*12.7*
31	343	<0.1	18	2	33.3	26.3	*13.4*
32	88	<0.1	19	2	32.6	26.6	*14.0*
33	28	<0.1	20	2	32.0	26.9	*14.6*
34	5	<0.1	22	3	31.3	27.2	15.3
35	2	<0.1	23	3	30.6	27.4	15.8

The table shows that for a false-positive rate $p_{fpr}$ of 0.05, we encounter reads that have, e.g., 20 (38, 20)-minimizers. Those reads have a chance of almost 40 % to have one false-positive count and a chance of almost 19 % to have two. This has a small effect on completely random hits. However, hits that match the query with more than the allowed errors could reach the threshold due to one or two false-positive minimizers.

To counter this, we introduce a correction term for the thresholds. We add a constant *c* to the threshold *t*(*x*), which is determined by increasing *c* until the probability $b_{p}(x,c)$ drops below a threshold $p_{max}$. For example, for $p=0.05$ and $p_{max}=0.15$, we have a correction of $+1$ for $14\le x\le 16$ and a correction of $+2$ for $17\le x\le 33$. For $p=0.02$, we have a correction of $+1$ for all *x*. The value for $p_{max}$ was experimentally determined using the benchmark from [12] such that the benchmark yielded no false positives and no false negatives. It is set to $p_{max}=0.15$. Those corrections are precomputed and incorporated into the thresholding.

#### Recommended choice of parameters

We want to free the user from setting internal parameters of the HIBF. Here, we describe how to set key parameters that are required. We assume that the user knows (1) how many *user bins* there are as input, (2) with how many errors or what kind of threshold the queries shall be searched. Wherever possible, we use sensible defaults, or infer parameters. For example, for constructing the index, we need to know the Bloom filter size. However, we can compute this parameter using the false-positive rate, which is a much more tangible parameter. When querying, we need to know the length of the queries. Likewise, we just compute this length if it is not provided.

1. Choice of *h* and $p_{fpr}$: Both the number of hash functions and the false-positive rate influence the allocated space for the HIBF. In our experiments, the values $h=2$ and $p_{fpr}=0.05$ turned out to be suitable for all analyzed data sets and are also used as default in Raptor. A lower $p_{fpr}$ limits the number of false-positive results that have to be handled downstream, while a higher $p_{fpr}$ can help to reduce memory consumption in cases where false-positive *k*-mers have little effect. For the inclined user, Thomas Hurst provides a website^5^ that visualizes the interaction of Bloom filter size, number of hash functions, and the false-positive rate.
2. Choice of $t_{max}$: In practice, the default of $\sqrt{\text {user bins}}$ works well and has a good space vs. query time tradeoff. It is also the default in Chopper. For more details, see the “The choice of *t*_*max*_” section.
3. Choice of *k*: Depending on the number of errors, *k* has to be chosen such that the *k*-mer lemma still has a positive threshold. For example, when querying reads of length 100 and allowing 4 errors, *k* has to be at most 20 ($100 - 20 + 1 - 4 \cdot 20 = 1$). Furthermore, *k* shall be such that a random *k*-mer match in the database is unlikely. For example, we chose $k=32$ for the RefSeq data set. In general, there is no drawback in choosing the (currently supported) maximum *k* of 32, as long as the aforementioned requirements are fulfilled.
4. Choice of *w*: In the case that minimizers should be used, *w* has to be bigger than *k*. Canonical *k*-mers are achieved with $w=k$. Depending on the choice of *k*, the choice of *w* has to be made such that we obtain positive thresholds with our probabilistic threshold. In general, we aim at having a minimum threshold of 3 for the (*w*, *k*) minimizers. Hence, we choose *w* as large as possible, such that the minimum threshold is 3. For example, this is obtained for 2 errors and read length 150 for (29, 20) minimizers, for read length 100 for (24, 20)-minimizers and for read length 250 for (40, 20) minimizers, which in turn reduces the amount of *k*-mers by factors of 5.5, 3, and 11, respectively.

**Table 3:** Used data

Dataset	Source	Link
Simulated data and queries	This paper, [29]	https://zenodo.org/record/7757110
RefSeq data	This paper, [30]	https://zenodo.org/record/7742011
RefSeq queries	This paper, [31]	https://zenodo.org/record/7741704
RefSeq result files	This paper, [32]	https://zenodo.org/record/7741886
RNA-Seq data	[33]	https://zenodo.org/record/1186393
RNA-Seq queries	This paper, [34]	https://zenodo.org/record/7752363

**Table 4:** Used Software. Each tool, if applicable, was compiled with optimizations enabled (-O3 -march=native)

Software	Source	Link	Version
Chopper	This paper	https://github.com/seqan/chopper	7837f1f
Raptor	[12]	https://github.com/seqan/raptor	560a45f
COBS	[10]	https://github.com/bingmann/cobs	1cd6df2
KMC	[38]	https://github.com/refresh-bio/KMC	v3.2.1
Metagraph	[13]	https://github.com/ratschlab/metagraph	v0.3.6
SeqOthello	[9]	https://github.com/LiuBioinfo/SeqOthello	68d47e0
HowDeSBT	[18]	https://github.com/medvedevgroup/HowDeSBT	379f4c8
Jellyfish	[39]	https://github.com/gmarcais/Jellyfish	v2.3.0
Squeakr	[40]	https://github.com/splatlab/squeakr	0d58134
Mantis	[5]	https://github.com/splatlab/mantis	b6979a2
Bifrost	[7]	https://github.com/pmelsted/bifrost	v1.2.0
Mason	[19]	https://seqan.de/apps/mason	b00001d
genome_updater	Vitor Piro	https://github.com/pirovc/genome_updater	v0.3.0

## Supplementary information

**Additional file 1.** Contains detailed hardware description, additional results and extended tables, and computation of the correction factor.

**Additional file 2.** Review history.

## Data Availability

All scripts and supplemental code used in this paper are published under the BSD 3-Clause License and available at https://github.com/seqan/raptor [28]. All used data is listed in Table 3. The applications *Chopper* (computes the HIBF layout) and *Raptor* are written in C++20 using the SeqAn Library [35], published under the BSD 3-Clause License, and available at https://github.com/seqan/chopper [36] and https://github.com/seqan/raptor [28], respectively. All software is listed in Table 4. The version of our source code used in this paper is further available at https://zenodo.org/record/7875008 [37].

## Footnotes

1 https://github.com/seqan/raptor/tree/main/util/Genome_Biology

2 https://github.com/Cyan4973/xxHash/tree/v0.7.3

3 https://github.com/hideo55/cpp-HyperLogLog/tree/517598b2

4 https://github.com/seqan/chopper/tree/7837f1/test/benchmark/benchmark_data/query_cost

5 https://hur.st/bloomfilter/
