# Supplementary material for: Hierarchical Interleaved Bloom Filter: enabling ultrafast, approximate sequence queries
Source: Genome Biol. 2023 May 31;24:131. doi: 10.1186/s13059-023-02971-4 (PMC10230713; doi:10.1186/s13059-023-02971-4)
Supplement: Supplementary file 1 — Additional file 1. Contains detailed hardware description, additional results and extended tables, and computation of the correction factor. [file 13059_2023_2971_MOESM1_ESM.pdf]

Hardware specifications

- Platform: Dell PowerEdge T640
- CPU:
  - Intel Xeon Gold 6248
  - 2 Sockets, 20 Cores per Socket, 2 Threads per Core
  - 40 physical CPUs, 80 logical CPUs (threads)
  - 3.9 GHz
- RAM:
  - 1 TiB
  - $2,666$ MHz
  - $16 \cdot$ HMAA8GR7AJR4N-WM (64 GiB each)

Results

Method	Pre-build Time	Pre-build RAM	Build Time	Build RAM	Build Index Size	Query Time	Query RAM	Statistics FP	Statistics FN	Statistics Accuracy
**20-mers**										
192-HIBF 1.5%	13:20	2.38 GiB	36:23	214.8 GiB	159.15 GiB	5:32	162.98 GiB	0.022 %	0	99.977 %
**24-mers**										
192-HIBF 1.5%	13:13	2.36 GiB	38:32	230.07 GiB	160.78 GiB	4:51	164.61 GiB	0.030 %	0	99.969 %
**28-mers**										
192-HIBF 1.5%	13:01	2.35 GiB	42:01	238.26 GiB	162.02 GiB	4:59	165.85 GiB	0.039 %	0	99.960 %
**32-mers**										
Mantis	140:35	1.4 GiB	2719:19	796.4 GiB	468.0 GiB	131:54	496.4 GiB	**0.0001** %	0.04851 %	99.951 %
COBS 5%	none		34:04	79.8 GiB	104.0 GiB	110:06	342.5 GiB	0.048 %	**0**	99.951 %
Metagraph	none		778:30	540.6 GiB	301.0 GiB	420:44	343.2 GiB	0.048 %	**0**	99.951 %
IBF 5%	none		**32:37**	293.1 GiB	292.5 GiB	13:08	296.3 GiB	0.048 %	**0**	99.951 %
IBF 1.5%	none		40:02	435.4 GiB	343.7 GiB	16:14	438.6 GiB	**0.047 %**	**0**	99.952 %
192-HIBF 5%	12:58	2.4 GiB	54:13	210.9 GiB	**109.9 GiB**	**4:55**	113.8 GiB	0.058 %	**0**	99.941 %
192-HIBF 1.5%	12:53	2.4 GiB	60:27	253.4 GiB	163.1 GiB	5:23	167.0 GiB	0.051 %	**0**	99.949 %
**(24,20)-minimizer, tau 0.99, pmax 0.4**										
Bifrost	none		1040:27	519.7 GiB	265.3 GiB	232:54	575.7 GiB	0.905 %	0.3239 %	98.770 %
IBF 5%	none		**11:24**	93.0 GiB	92.3 GiB	4:19	96.2 GiB	0.113 %	0.0003 %	99.886 %
IBF 1.5%	none		12:01	137.9 GiB	137.3 GiB	6:23	141.1 GiB	**0.112 %**	**0.0003 %**	99.887 %
192-HIBF 5%	13:36	2.4 GiB	12:55	57.23 GiB	**34.4 GiB**	**1:48**	38.3 GiB	0.128 %	0.0003 %	99.871 %
192-HIBF 1.5%	13:23	2.4 GiB	15:23	78.37 GiB	51.1 GiB	1:50	54.9 GiB	0.116 %	0.0003 %	99.882 %
**(32,20)-minimizer, tau 0.99, pmax 0.4**										
192-HIBF 1.5%	13:28	2.36 GiB	5:39	31.43 GiB	22.14 GiB	1:04	25.97 GiB	0.302 %	0.00003 %	99.698 %
**(40,20)-minimizer, tau 0.99, pmax 0.4**										
192-HIBF 5%	12:55	2.35 GiB	3:56	15.80 GiB	**9.62 GiB**	2:49	13.45 GiB	0.465 %	0.00001 %	99.534 %
192-HIBF 1.5%	13:34	2.36 GiB	4:04	20.66 GiB	15.11 GiB	**1:07**	18.94 GiB	0.452 %	0.00001 %	99.547 %
**(28,24)-minimizer, tau 0.99, pmax 0.4**										
192-HIBF 1.5%	13:10	2.36 GiB	12:12	73.88 GiB	51.59 GiB	1:31	55.42 GiB	0.115 %	0.0004 %	99.884 %
**(32,24)-minimizer, tau 0.99, pmax 0.4**										
192-HIBF 1.5%	13:08	2.35 GiB	7:31	43.01 GiB	30.87 GiB	1:11	34.69 GiB	0.229 %	0.00003 %	99.770 %
**(40,32)-minimizer, tau 0.99, pmax 0.4**										
192-HIBF 1.5%	13:06	2.36 GiB	7:28	35.32 GiB	31.49 GiB	1:09	35.32 GiB	0.242 %	0.00001 %	99.758 %

Table S1 All complete genomes of Archaea and Bacteria in RefSeq. The uncompressed data set has a size of about 98.8 GiB. Query reads of length 250 bp were simulated using the Mason simulator [1]. Mantis does not support minimizers and could only be used with $k = 32$ because it crashed for $k = 20$. Although Mantis is technically an exact method outputting k-mer counts, a threshold (in this case 0.7) needs to be applied to determine the query membership resulting in few false positives/negatives (see Section Mantis thresholding). Bifrost was run with $(24, 20)$-minimizers and a threshold of 0.36. For details on the chosen thresholds, see Section Validation.

All measurements used to produce Figure 6 can be found in Table S1.

RNA-Seq data

In addition to the artificial data and the RefSeq benchmark, we also performed benchmarks on RNA-Seq data.

This real-world data set consists of $1,742$ RNA-Seq files and is a subset of the data used as a benchmark in similar applications [2]. Similar to [3], experiments

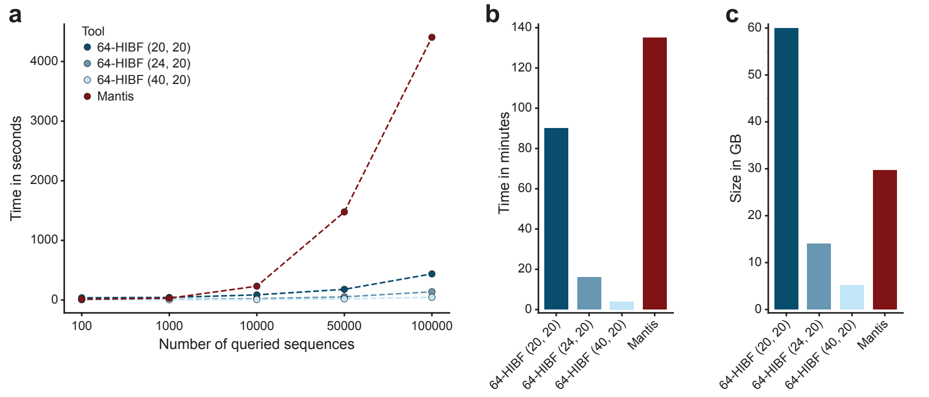

Figure S1 RNA-Seq results. (a) The query time in seconds for the HIBF and Mantis for 100/1,000/10,000/50,000/100,000 transcripts using a threshold of 0.8. (b) The build time in minutes. (c) The index size in GiB.

with a read length below 50 bp were excluded. Low frequency k-mers, which are likely the result of sequencing errors, are excluded by applying the same cutoffs as [2]. Unlike previous analysis, this analysis was performed with one thread to stay consist with previous analysis [2, 3]. Since the benefits of the HIBF over the IBF were shown in the previous sections, we focused on the comparison of the HIBF against its competitor *Mantis*.

For this data set, the HIBF can be built faster than Mantis for any setting. The resulting index is larger than that of Mantis for k-mers and smaller for $(24, 20)$-minimizers as well as $(40, 20)$-minimizers (see Figure S1). The larger index compared to Mantis is expected because the RNA-Seq data set is highly redundant, and Mantis's color classes apply a compression scheme that is based on these similarities. In contrast, the HIBF stores all k-mers explicitly. When the dataset is not highly redundant, as is the case for metagenomic data, the HIBF index is significantly smaller than Mantis.

Furthermore, we compared the query performance of Mantis and the HIBF by searching for $100/1,000/10,000/50,000/100,000$ random transcripts from the human genome with a threshold of 0.8. As can be seen in Figure S1a, the HIBF outperforms Mantis by orders of magnitude once the number of queries is greater than $1,000$. With fewer queries, only the HIBFs with $(24, 20)$-minimizers and $(40, 20)$-minimizers are faster than Mantis. This is due to the index loading time, which is constant for each query call, but influences the overall query time less the more queries are conducted at once.

The RefSeq data set

The data set containes all RefSeq Archaea and Bacteria Complete Genomes as of 28-01-2022, with a size of 98.8 GiB uncompressed and 28.5 GiB compressed. The sequences are in the FASTA format. The data set has $25,321$ files and $54,348$ FASTA records.

Method	Build		Query 1000		Query 1 Million		Query 5 Million		Query 10 Million	
	Time	RAM	Time	RAM	Time	RAM	Time	RAM	Time	RAM
COBS	00:34:04	104	00:01:15	106.02	00:11:34	127.47	00:57:13	222.91	01:50:02	342.51
Bifrost	17:46:25	68	01:17:43	282.89	01:21:55	282.89	01:39:00	292.89	02:02:33	282.89
Metagraph	56:36:22	216	00:03:46	231.42	00:13:24	249.02	00:52:09	249.56	01:37:50	254.97
SeqOthello	49:26:04	315	00:29:34	30.70	01:43:48	287.71	NA	NA	NA	NA
Mantis	45:19:00	496	00:07:27	468.55	00:18:47	470.67	01:05:47	472.93	02:18:33	496.40
IBF	00:32:37	374	00:02:31	374.04	00:03:59	374.38	00:08:28	376.83	00:13:08	379.83
HIBF	00:38:28	133	00:01:01	133.05	00:01:15	132.70	00:02:29	134.57	00:04:11	136.47

Table S2 Raw data for Figure 5. All complete genomes of Archaea and Bacteria in RefSeq. The uncompressed data set has a size of about 98.8 GiB. Ten million query reads of length $250\,bp$ were simulated using the *Mason simulator* [1]. The parameters used for all tools (if applicable) were: canonical k-mers $k = 32$, no k-mer filtering, false positive rate $5\,\%$, 2 hash functions, 32 threads, query search threshold 0.7.

Mantis thresholding

In contrast to other tools compared to in this work, Mantis does not provide the feature of applying a threshold to its k-mer counts. Instead, it outputs counts for every query and every indexed sample. The counts arise from the **unique** k-mers of a query. This poses a problem for a general threshold, since the threshold is always relative to the total number of k-mers, i.e., **all** k-mers. We applied a threshold according to the *k-mer lemma* [4]. For example, for queries of length 250, there are $250 - 32 + 1 = 219$ 32-mers per query. When searching those queries with up to 2 errors, the *k-mer lemma* states that at least 155 k-mers need be found, which corresponds to a threshold of $\lfloor\frac{155}{219}\rfloor = 0.7$. However, using unique k-mers shrinks the number of k-mers, i.e., there may be fewer than 219 k-mers in the query. Thus, the threshold is less tight, resulting in the possibility of false classifications in the form of false negatives and false positives.

Statistics of different values for t_{max} for the RefSeq data set

We computed statistics with the tool *Chopper* on this data set to choose a fitting t_{max}. The results are shown in table S3 (*query cost*, *space cost*, and *total cost*). We validated the statistics by actually building and querying the respective HIBF (*real time*, *real mem*, and *real total*).

t_{max}	**64**	**128**	**sq=192**	**256**	**512**	**1024**	**2048**	**4096**	**8192**
bin penalty	1.00	1.01	1.09	1.17	1.34	1.83	2.70	5.39	8.62
query cost	1.00	0.84	**0.78**	0.83	1.06	1.23	1.51	2.26	3.59
space cost	1.00	0.79	**0.68**	**0.68**	0.72	0.80	0.90	0.93	0.84
total cost	1.00	0.66	**0.53**	0.56	0.76	0.98	1.36	2.10	3.01
real time	1.00	0.85	0.62	0.58	0.59	0.72	0.94	1.48	2.38
real mem	1.00	0.79	0.64	0.73	0.69	0.81	0.90	0.97	0.76
real total	1.00	0.67	0.39	0.42	0.40	0.59	0.84	1.43	1.80

Table S3 Expected relative runtimes, memory consumption and the runtime relative to memory consumption for different choices of $\mathbf{t_{max}}$ and $\mathbf{p_{fpr} = 0.0125}$. *bin penalty* refers to the increase in runtime when querying an original IBF with t_{max} bins. All numbers are given as ratios compared to the (H)IBF version with 64 (user) bins. *query, space* and *total cost* refers are estimated cost of an t_{max}-HIBF computed by the tool *Chopper*. *real time, real mem* and *real total* refer to the experimentally derived ratios of an t_{max}-HIBF using (24,20)-minimizer, 4 hash functions and an $p_{fpr} = 0.0125$. The minimum time is reached for $t_{max} = 192$. The minimum relative run time is also reached for $t_{max} = 192$, followed by the value for $t_{max} = 256$.

Determining the correction factors

Let p_{fpr} be the desired false positive rate, and p_{corr} the false positive rate such that conducting s simultaneous tests yields a false positive rate of p_{fpr}. Then p_{corr} can be computed as follows:

$$\begin{aligned} p_{fpr} &= 1-(1-p_{corr})^s \\ \Leftrightarrow \quad 1-p_{fpr} &= (1-p_{corr})^s \\ \Leftrightarrow \quad \ln(1-p_{fpr}) &= s\cdot\ln(1-p_{corr}) \\ \Leftrightarrow \quad \frac{\ln(1-p_{fpr})}{s} &= \ln(1-p_{corr}) \\ \Leftrightarrow \quad e^{\frac{\ln(1-p_{fpr})}{s}} &= 1-p_{corr} \\ \Leftrightarrow \quad p_{corr} &= 1-(1-p_{fpr})^{\frac{1}{s}} \end{aligned} \tag{1}$$

Indeed, multiple testing using the false positive rate p_{corr} yields a false positive rate (p'') equal to p_{fpr}:

$$\begin{aligned} p'' &= 1-(1-p_{corr})^s \\ p'' &= 1-(1-(1-(1-p_{fpr})^{\frac{1}{s}}))^s \\ p'' &= 1-((1-p_{fpr})^{\frac{1}{s}})^s \\ p'' &= 1-(1-p_{fpr}) \\ p'' &= p_{fpr} \quad \square \end{aligned} \tag{2}$$

The correction factor is now computed as the ratio of the Bloom filter size with p to the Bloom filter size with p_{corr}. Recall the formula of the Bloom filter size:

$$m(p) \approx -\frac{h\cdot n}{\ln(1-p^{\frac{1}{h}})} \tag{3}$$

We want to compute $f_{corr} = m(p_{corr})/m(p_{fpr})$:

$$\begin{aligned} f_{corr} &= \frac{\frac{-h\cdot n}{ln(1-p_{corr}^{1/h})}}{\frac{-h\cdot n}{ln(1-p_{fpr}^{1/h})}} \\ f_{corr} &= \frac{-h\cdot n}{ln(1-p_{corr}^{\frac{1}{h}})}\cdot\frac{ln(1-p_{fpr}^{\frac{1}{h}})}{-h\cdot n} \\ f_{corr} &= \frac{\ln(1-p_{fpr}^{\frac{1}{h}})}{\ln(1-p_{corr}^{\frac{1}{h}})} \end{aligned} \tag{4}$$

References

1. Holtgrewe M. Mason – A Read Simulator for Second Generation Sequencing Data. Technical Report FU Berlin. 2010 Oct. Available from: http://publications.imp.fu-berlin.de/962/.
2. Pandey P, Almodaresi F, Bender MA, Ferdman M, Johnson R, Patro R. Mantis: A Fast, Small, and Exact Large-Scale Sequence-Search Index. Cell Syst. 2018 aug;7(2):201-7.e4. Available from: https://linkinghub.elsevier.com/retrieve/pii/S2405471218302394papers3://publication/doi/10.1016/j.cels.2018.05.021https://doi.org/10.1016/j.cels.2018.05.021.
3. Seiler E, Mehringer S, Darvish M, Turc E, Reinert K. Raptor: A fast and space-efficient pre-filter for querying very large collections of nucleotide sequences. iScience. 2021;24(7):102782. Available from: https://www.sciencedirect.com/science/article/pii/S2589004221007501.

4. Jokinen P, Ukkonen E. Two algorithms for approxmate string matching in static texts. In: Tarlecki A, editor. Mathematical Foundations of Computer Science 1991. Lecture Notes in Computer Science. Springer Berlin Heidelberg; 1991. p. 240-8.
