# Supplementary material for: Hierarchical Interleaved Bloom Filter: enabling ultrafast, approximate sequence queries
Source: Genome Biol. 2023 May 31;24:131. doi: 10.1186/s13059-023-02971-4 (PMC10230713; doi:10.1186/s13059-023-02971-4)
Supplement: Supplementary file 2 — Additional file 2. Review history. [file 13059_2023_2971_MOESM2_ESM.docx]

**1^st^ round**

**Reviewer 1**

The manuscript describes a data structure able to efficiently partition kmers from large datasets collection in a way allowing fast and memory efficient indexing.

Using this structure implementation allows very resource efficient indexing and querying of very large databases compromising thousands datasets.

The methods are well described and present very interesting inputs to the field, the improvements over Raptor are impressive and well validated demonstrating the interest of the approach.

Moreover since it was integrated to the seqan library the implementation chopper is easy to install, however the usage cannot be yet described as easy and could be improved with example or use-cases.

The experiments are clearly described and could be easily replicated, making benchmarks scripts available would be a plus.

However, the manuscript failed to present a complete state of the art and only compare itself with some exact methods (Bifrost and Mantis).

Compared a probabilist approach to an exact one is not fair and a very large spectrum of probabilistic method does exist.

SBT (Solomon, Brad, and Carl Kingsford. "Improved search of large transcriptomic sequencing databases using split sequence bloom trees." Journal of Computational Biology 25.7 (2018): 755-765.), SSBT,ALLsomeSBT(Sun, Chen, et al. "Allsome sequence bloom trees."International Conference on Research in Computational Molecular Biology. Springer, Cham, 2017.), HowDeSBT(Harris RS, Medvedev P. 2020. Improved representation of sequence bloom trees. Bioinformatics 36: 721-727. doi:10.1093/bioinformatics/btz662) for tree like approach, Bigsie(Bradley P, den Bakker HC, Rocha EP, McVean G, Iqbal Z. 2019. Ultrafast search of all deposited bacterial and viral genomic data. Nat Biotechnol37: 152-159. doi:10.1038/s41587-018-0010-1), Cobbs(Bingmann T, Bradley P, Gauger F, Iqbal Z. 2019. COBS: a COmpact Bit-sliced Signature index. In Proceedings of the Twenty-sixth Int'l Symposium on String Processing and Information Retrieval, Segovia, Spain, pp. 285−303.) PAC(Marchet, Camille, and Antoine Limasset. "Scalable sequence database search using Partitioned Aggregated Bloom Comb-Trees." bioRxiv (2022).) and Metagraph (Karasikov, Mikhail, et al. "Metagraph: Indexing and analysing nucleotide archives at petabase-scale." BioRxiv (2020).) for matrices approaches and SeqOthello (Yu, Ye, et al. "SeqOthello: querying RNA-seq experiments at scale." Genome biology 19.1 (2018): 1-13.) for MPHF approach as presented in Marchet, Camille, et al. "Data structures based on k-mers for querying large collections of sequencing data sets." Genome Research 31.1 (2021): 1-12.

A benchmark including at least some probabilistic methods seems necessary with some consideration on the impact on indexing only minimizers instead of storing all kmers.

Another to include the state of the art would be to show of Chopper can improve some other methods than Raptor.

Lastly, the qualitative results based on false positives and negatives could be better described as it is confusing to see exact method like Mantis displaying false positives and negative and probabilistic methods presenting zero errors (from the random genomes experiment).

**Reviewer 2**

The manuscript presents HIBF, a new data structure for indexing k-mers in collections of samples. This is now a popular problem in bioinformatics, with many solutions being proposed (and reviewed in Marchet et al, 2021, Genome Res, which should be cited in this manuscript). The method appears interesting, though there are unnoted commonalities with COBS, which also conceptually interleaves Bloom filters and introduces a mechanism to deal with datasets of unbalanced sizes. The methods are well-described, and the DP layout algorithm is interesting. I could test the software on the provided example data and it worked well. In the current state of the manuscript, it is challenging to estimate the merit and novelty of HIBF, as the benchmark dataset size is small, the compared methods are outdated, and novelty with respect to COBS is not explained.

Major remarks:

* The compared methods are not state of the art. MetaGraph, pre-printed in 2020, should definitely have been included, as it is currently the best-performing indexing method available. A few other methods are also noted below.
* The scale of the benchmarks is not convincing enough, given that only 98 GB of reference genomes were indexed at most. Such a data size is so small that even BLAST/DIAMOND can easily process it, with more versatile alignment features. At the very least, benchmarks should be at the scale of tens to hundreds of terabytes to show any novelty. COBS benchmark was over 4 TB of data, Bentley-Saxe Mantis was 110 TB of FASTQs, the RAMBO tool indexed 170 TB of data.
* The cited version of Mantis has since been superseded by two other : Mantis-MST and Bentley-Saxe Mantis (https://www.ncbi.nlm.nih.gov/pmc/articles/PMC7185321/ and https://academic.oup.com/bioinformatics/article-abstract/38/12/3155/6553005?redirectedFrom=fulltext&login=false) Which version was used in this manuscript?
* According to the COBS paper (https://arxiv.org/pdf/1905.09624.pdf), COBS is significantly faster than Mantis, for both construction and query. Therefore it would be important to also add COBS to the benchmark.
* The main methodological novelty of HIBS compared to COBS is unclear. COBS also conceptually interleaves Bloom filters, and introduces a mechanism to deal with unbalanced dataset sizes.
* It is also unclear why HowDeSBT and SeqOthello are not mentioned nor compared to, both could potentially be competitive with the method here.

Minor remarks:

* It would be beneficial to indicate early on why the DP layout strategy is not a straightforward bin packing.
* Figure 3, IBF-2 caption should be "2 TBs" not "3 TBs".
* Typos: ", a number is infeasible for previously published tools"

**Authors’ response**

R1: Moreover, since it was integrated to the SeqAn library, the implementation chopperis easy to install. However, the usage cannot be yet described as easy and could beimproved with example or use-cases. The experiments are clearly described and couldbe easily replicated, making benchmarks scripts available would be a plus.

Answer:

The benchmarking scripts were available. However, they were indeed difficult to find.

We made our scripts more prominent by mentioning (and linking) them in themanuscript. All scripts can be found on our GitHub page

(https://github.com/seqan/raptor/tree/main/util/Genome_Biology).

Furthermore, we added Chopper to Raptor such that users only need to install onetool. We have added tutorials to Raptor on how to use the (H)IBF (https://seqan-raptor.vercel.app).

=====================================================================

R1: However, the manuscript failed to present a complete state of the art and onlycompare itself with some exact methods (Bifrost and Mantis).

Compared a probabilistic approach to an exact one is not fair, and a very largespectrum of probabilistic method does exist:

SBT https://doi.org/10.1089/cmb.2017.0265

ALLsomeSBT https://doi.org/10.1089/cmb.2017.0258

HowDeSBT https://doi.org/10.1093/bioinformatics/btz662

Bigsie https://doi.org/10.1038/s41587-018-0010-1

Cobs https://doi.org/10.1007/978-3-030-32686-9_21

PAC https://doi.org/10.1101/2022.02.11.480089

Metagraph https://doi.org/10.1101/2020.10.01.322164

SeqOthello https://doi.org/10.1186/s13059-018-1535-9

MUST CITE: "Data structures based on k-mers ..."

https://doi.org/10.1101/gr.260604.119

A benchmark including at least some probabilistic methods seems necessary, withsome consideration on the impact on indexing only minimizers instead of storing all k-mers.

R2: The compared methods are not state-of-the-art. MetaGraph, pre-printed in 2020,should definitely have been included, as it is currently the best-performing indexingmethod available. A few other methods are also noted below.....

According to the COBS paper, COBS is significantly faster than Mantis, for bothconstruction and query. Therefore, it would be important to also add COBS to thebenchmark.

It is also unclear why HowDeSBT and SeqOthello are not mentioned nor compared to,both could potentially be competitive with the method here.

Answer:

We have installed and tried to run the following tools:

Metagraph, COBS, SeqOthello, PAC, HowDeSBT, and kmtricks (HowDeSBT’ssuccessor).

We added COBS, Metagraph, and SeqOthello to the RefSeq data set analysis. Wewere unable to run PAC and kmtricks on the RefSeq data set. We contacted theauthors of both tools to troubleshoot possible errors, but, so far, could not make thetools run on this data set.

=====================================================================

R2: The scale of the benchmarks is not convincing enough, given that only 98 GB ofreference genomes were indexed at most. Such a data size is so small that evenBLAST/DIAMOND can easily process it, with more versatile alignment features. At thevery least, benchmarks should be at the scale of tens to hundreds of terabytes to showany novelty. COBS benchmark was over 4 TB of data, Bentley-Saxe Mantis was 110TB of FASTQs, the RAMBO tool indexed 170 TB of data.

Answer:

In the supplement, we have (and had already) included a benchmark on the RNA-Seqdataset of 4 TiB, which is the same as used in other works. Notably, the RNA-seq datasets indexed in the Mantis and Metagraph paper are heavily preprocessed beforeindexing. Thus, the actual amount of k-mers stored is way less than the input size. Incontrast, our RefSeq data set is not filtered, and hence the input is proportional to thek-mer content that needs to be stored.

We would like to add that a comparison on larger data (e.g., full RefSeq) would scale,but would take much longer, considering the build time of other tools. In addition, we donot have the same resources as, for example, Mantis. In their most recent paper,Mantis preprocessed their 40,000 samples on a cluster with 150 nodes (machines) infour weeks. On a single machine, this process would take around 11.5 years.

We have started downloading and processing those 40,000 files during theresubmission. We can report that we indexed a subset of the RNA-Seq data (10,000samples, 22 TiB), preprocessed with the same cutoffs as Mantis, in about 350 GiB,which shows that our approach scales.

=====================================================================

R2: The cited version of Mantis has since been superseded by two other: Mantis-MSTand Bentley-Saxe Mantis. Which version was used in this manuscript?

Answer: Thank you for pointing it out. This was a citation error. We use Mantis-MST(https://doi.org/10.1089/cmb.2019.0322). The most recent paper(https://doi.org/10.1093/bioinformatics/btac142) adds dynamization, which we did notneed.

=====================================================================

R2: The main methodological novelty of HIBF compared to COBS is unclear. COBSalso conceptually interleaves Bloom filters, and introduces a mechanism to deal withunbalanced dataset sizes.

Answer:

We thank the reviewers for pointing out the similarity between these methods. Weincluded a short comparison in the background section where we introduce the tools.Briefly, COBS stores a matrix of Bloom filters, while the IBF is a single bitvectorrepresenting multiple Bloom filters. COBS merges the input in consecutive bins andbuilds subindices that are queried sequentially. In contrast, the HIBF also splits largebins and creates a hierarchical structure to avoid querying all subindicies.

=====================================================================

R2: Another thing would be to show if Chopper can improve some other methods thanRaptor.

Answer: We fully agree that this could be beneficial for other methods, for example,COBS. However, this was not the scope of the current paper and is subject to furtherinvestigations by our group. It will be considered together with other versions ofChopper.

=====================================================================

R1: Lastly, the qualitative results based on false positives and negatives could bebetter described as it is confusing to see exact method like Mantis displaying falsepositives and negative and probabilistic methods presenting zero errors (from therandom genomes experiment).

Answer: We thank the reviewer for pointing out the confusing nature of false negativesand false positives regarding exact methods. We adapted the manuscript to betterexplain what we mean with FP/FN and how they come to be with an exact method like mantis. A whole section in the supplement now explains the thresholding for Mantis,which is referenced in the result section on accuracy. Briefly, Mantis does not, incontrast to all other tools, provide thresholding. The final results are k-mer counts.However, Mantis counts the unique k-mers of the query instead of all k-mers. Forexample, if a k-mer occurs twice in a query, it is only counted once. This makesthresholding based on a single cutoff, such as provided by the k-mer lemma, verydifficult. Hence, some queries may lead to false answers for Mantis.

=====================================================================

R2 (minor): It would be beneficial to indicate early on why the DP layout strategy is nota straightforward bin packing.

Answer: We have added a sentence when describing the main idea of the HIBF at thebeginning of the Results section that we hope clears the issue.

“This distribution of user bins cannot be solved by a simple bin packing algorithmbecause of two reasons: 1) user bins can share k-mers, making the problem similar tothe more complex VM packing variant, and 2) splitting the content of user binsincreases the complexity further and an applicable solution in the context of sequenceanalysis was not proposed as far as we know.”

**2^nd^ round**

**Reviewer 1**

Based on my review, I am pleased to confirm that the authors have made satisfactory modifications to their paper.

**Reviewer 2**

I have seen the revision and I am satisfied by the responses. The authors provided new benchmarks that answered my concerns regarding the position of HIBF with respect to the state of the art. With this more comprehensive benchmark, I am more confident that HIBF is a valuable contribution. Last minor point: figure 3 top right has a character that renders oddly in my pdf preview: “?seq” instead of “>seq”. I trust this will be fixed during proof stage.
